# Introducing information literacy and careers in librarianship to New York City high school students: a case report

**DOI:** 10.5195/jmla.2026.2354

**Published:** 2026-07-01

**Authors:** Andy Hickner, Diana Delgado, Jessica Boyle, Lynne Holden, Sarah Jewell, Michael A. Wood, Terrie R. Wheeler

**Affiliations:** 1 alh4014@med.cornell.edu Education and Outreach Librarian, Samuel J. Wood Library & C.V. Starr Biomedical Information Center, Weill Cornell Medicine, New York, NY; 2 did2005@med.cornell.edu, Associate Director, Information, Education and Clinical Services, Samuel J. Wood Library & C.V. Starr Biomedical Information Center, Weill Cornell Medicine, New York, NY; 3 jrottenstein@gmail.com, Science Teacher, Knowledge and Power Preparatory International Academy, Bronx, NY; 4 holden@medicalmentor.org, Founder and Chief Executive Officer, Medical Mentor Institute, New York, NY; 5 stjewell@gmail.com, Chief Librarian and Associate Dean, City University of New York School of Medicine, New York, NY; 6 mawood@med.cornell.edu, Assistant Librarian, Resource Management, Samuel J. Wood Library & C.V. Starr Biomedical Information Center, Weill Cornell Medicine, New York, NY; 7 tew2004@med.cornell.edu, Library Director, Samuel J. Wood Library & C.V. Starr Biomedical Information Center, Weill Cornell Medicine, New York, NY

**Keywords:** Literacy, Careers, New York, New York City, High Schoolers

## Abstract

**Background:**

Black, Indigenous, and People of Color (BIPOC) are underrepresented in librarianship. We developed a curriculum to introduce high school students from BIPOC communities to careers in health sciences librarianship and to concepts and skills in health information literacy.

**Case Presentation:**

Librarians at Weill Cornell Medicine partnered with Mentoring in Medicine, a Bronx-based nonprofit, to develop the Community Health Ambassador Program (CHAmP) curriculum. The project settings were at three New York City schools. The project took place over two years, with the pilot taking place in year 1, followed by an assessment which included data from pre- and posttests measuring student learning and focus groups with the students to gather feedback about their experience. Informed by data from the year one assessment, we worked with an experienced high school teacher to redesign the curriculum for year 2. Changes included reducing the amount of lecture and providing more time for activities to reinforce the content, including a team final project and presentations. In year two focus groups, students demonstrated increased understanding of health sciences librarianship and indicated increased engagement and enjoyment of the course compared to year 1. There was a statistically significant improvement in the mean student score from the pretest to the posttest.

**Conclusions:**

The revised curriculum resulted in increased student engagement and statistically significant improvement in learning compared to the year one pilot. We have published the full curriculum online under a Creative Commons license so that other organizations may implement it in their communities.

## BACKGROUND

Statistical reports show Black, Indigenous, and People of Color (BIPOC) individuals are poorly represented in librarianship and library leadership. As of 2024, according to the United States (US) Bureau of Labor Statistics, only 6.7% of US librarians were Black or African, 4% Asian, and 13.1% Hispanic or Latino, and more than 82% of US librarians were White [1]. Lack of medical workforce diversity impacts health equity; racial concordance between patients and providers can improve patient experiences [2] as well as patient-reported clinical quality of care [3]. In addition, studies indicate health literacy is associated with adolescents’ health promoting behaviors, physical health outcomes, and high-risk behaviors [4,5]. Health literacy is defined as the “cognitive and social skills which determine the motivation and the ability of individuals to gain access to, understand and use information in ways which promote and maintain good health” [6]. In a randomized clinical trial published in 2023, Black adults had significantly more trust in receiving health information from racially concordant presenters, whether their health literacy levels were adequate or limited [7].

Many curricula focused on careers in health professions and health information literacy have been targeted to high school students. Most students who choose STEM (science, technology, engineering and medicine) careers do so in high school [8], and students who report an interest in a science career in eighth grade are (unsurprisingly) more likely to graduate with a degree in the sciences [9]. School-based health literacy programs are typically targeted toward adolescents (13-18 years) [10], and librarian-led outreach program was found to have improved high school students’ information evaluation skills and their knowledge and interest in health careers [11]. This evidence suggests K-12 as an appropriate level to introduce students to a curriculum combining health information literacy and the career of health sciences librarianship. We leveraged a relationship with a local organization with an established presence in New York City high schools to develop, pilot, and evaluate such a curriculum.

## CASE PRESENTATION

Located in New York city, Weill Cornell Medicine (WCM) is an academic medical center founded in 1898. WCM’s core mission focuses on delivering exceptional and individualized patient care, advancing innovative research, and providing exemplary education [12]. WCM has over 13,000 faculty and staff and 1,818 students and from 2024-2025 had an estimated 3.58 million patient encounters [13]. The Samuel J. Wood Library (Wood Library), which opened in 1962, is a hub for research support, electronic resources, and physical space [14]. The Wood Library supports the evolving clinical services, research, and medical education needs of WCM, NewYork-Presbyterian Hospital and affiliated institutions. Additionally, the Wood Library oversees the Myra Mahon Patient Resource Center (MMPRC), which is dedicated to providing patients, families, and caregivers with credible evidence-based information to support healthcare decision making [15].

With grant funding (RE-254913-OLS-23) from the US Institute for Museum and Library Services (IMLS), we established the Community Health Ambassador Program (CHAmP) with three aims: (1) increasing the number of high school students from diverse and underrepresented backgrounds who are interested in health sciences librarianship, (2) teaching youth how to combat health-related misinformation and disinformation, and (3) teaching health information skills in order to combat health disparities and improve health literacy in underserved communities.

Our project partner was Mentoring in Medicine (MIM), a nonprofit Bronx-based educational youth development program. MIM’s mission is to inspire and equip students from underrepresented backgrounds to pursue health and science careers. MIM has developed school-based and after-school programs, which are currently in thirty middle and high schools in NYC and five in Long Island [16]. Through our partnership with MIM we were able to insert our curriculum into the anatomy class of tenth, eleventh, and twelfth grade students during their normal class time for two and a half weeks of forty-five-minute class periods. The anatomy class was selected because it is where MIM programs are typically embedded take place during the school day.

Our team consisted of WCM Wood librarians and MIM leaders and teachers with expertise in instructional design and health literacy. The selected Wood Librarians brought extensive experience in various areas, including diversity, equity, and inclusion (DEI) initiatives. Several have served on WCM, regional, and national committees, as well as on career pipeline programs. The librarians have also been instrumental in developing inclusive policies within the Wood Library. Additionally, the librarians possessed skills in health literacy education and have experience teaching at the high school, undergraduate, and adult education levels. The librarians’ experience also includes education design and instructional delivery. Multiple BIPOC professionals were represented on our team. We were also supported by an Advisory Board of BIPOC health sciences librarians and professors, whose purpose was to provide external feedback and review of the curriculum.

WCM Human Research Compliance reviewed our study protocol and determined that it did not constitute human subjects research and therefore was not subject to review by the Institutional Review Board (#22-09025294).

### Year One Pilot

Our team developed the pilot curriculum in summer and fall 2023 with the input of an experienced high school teacher (JB). The curriculum consisted of five modules plus a performance task, which was a group final project that the students presented to the instructors and their classmates on the last day of class as a celebration of learning. Each module was taught by a different team member, who designed their module and developed corresponding instructional materials, and included a pre-recorded video by an Advisory Board member about how they chose librarianship as a career. Librarian instructors solicited feedback on their proposed instructional plans and materials from the MIM team members in weekly team meetings.

We piloted the curriculum in February 2024 at two New York City schools: Mount Saint Michael Academy (MSMA) in the Bronx, and Thurgood Marshall Academy for Learning and Social Change (TMA) in Harlem. A total of 45 students participated across the two sites. Both schools have majority Black or Hispanic non-white student bodies [17,18]. TMA is coeducational while MSMA is all boys.

On the first day of class, students took a pretest which measured their baseline knowledge of health literacy, followed by an identical posttest on the last day of class. Group final project presentations, evaluated by instructors using a five-item scoring rubric, comprised a second measure of student learning. MIM student volunteers conducted post-intervention focus groups at both schools after the final day of class to gather student feedback about the course, with a secondary purpose of assessing their retention of concepts and course material.

Contrary to expectations, posttest scores declined compared to pretest scores (Table One), although the decline in average score was not statistically significant, with a two-tailed p-value of 0.0612. This was mostly driven by a 19% drop in average scores at one school. In the pretest, no scores fell below 5 (out of 10) at either school. In the posttest, there were seven scores below 5, most of which were from one school. Low scores (2 or 3) correlated with rapid completion times (37 and 79 seconds), compared to an average of 256 seconds, suggesting several students at one school “blew off” the posttest since it was ungraded.

**Table 1:** Year One Pre-Posttest Scores by Question

Question	MSM pretest # correct responses (N=21)	MSM pretest percentage correct (N=21)	MSM posttest # correct responses (N=22)	MSM posttest percentage correct (N=22)	TMA pretest # correct responses (N=23)	TMA pretest percentage correct (N=23)	TMA posttest # correct responses (N=22)	TMA posttest percentage correct (N=22)
When assessing the authority of a source's author, which factors should be taken into account?	19	90%	19	86%	22	96%	20	91%
When assessing the purpose of a source, which aspects should be taken into consideration?	20	95%	14	64%	22	96%	19	86%
Which of the following best defines health literacy?	17	81%	12	55%	17	74%	19	86%
You conduct a community survey about diabetes to see if the community answers right or wrong. The following table represents the answers to that survey. Which of the following bar graphs accurately represents your results?	18	86%	17	77%	19	83%	19	86%
An account you follow on TikTok shares a video where they interview a famous doctor about a special diet they are selling to help people shed fat quickly while adding muscle. Which of the following is most true?	18	86%	17	77%	20	87%	15	68%
Which statement best defines misinformation?	12	57%	16	73%	15	65%	10	45%
What is the purpose of fact-checking a news story?	19	90%	16	73%	20	87%	18	82%
Which of the following may indicate a news article is biased?	20	95%	17	77%	19	83%	17	77%
Popular literature typically has which of the following characteristics?	9	43%	13	59%	9	39%	16	73%
In addition to the URL, what would a citation need to include to make an online news article findable?	16	76%	14	64%	16	70%	16	73%

TMA = Thurgood Marshall Academy, MSM = Mount Saint Michael Academy

In post-intervention focus groups, students said they felt there was too much lecture and that the course was not interesting or enjoyable. According to students, the course felt disjointed, with a lack of connection or reinforcement between topics. Students overwhelmingly indicated they were still not interested in librarianship. In addition to the issues described by the students, there was some miscommunication and misunderstanding regarding the availability of technical support, which was problematic as the curriculum involved extensive use of the projector. This resulted in reduced teaching time due to technical troubleshooting.

### Year Two: Revised curriculum

Based on our findings from the pilot assessment, we revised the curriculum for year two. The year two curriculum was offered in November and December 2024. The high school teacher on our team (JB) led this process and redesigned the course, leveraging her expertise derived from many years of experience teaching high school students. We revised course and session learning outcomes to enhance cohesiveness across the modules, streamline the curriculum, reduce lecture time, and allow more time for activities. We sought to integrate content through task-based learning and reduce students’ cognitive load. We also rewrote the performance task. In the new task, students played librarians hired by a celebrity to stop the spread of online misinformation about their diagnosis. This prompt provided a “hook” for the subject matter and offered students choice of which celebrity to focus on.

Table Two shows the key changes to content included rolling the year one module “How to read news articles” into the “Find and Identify Credible Health Information” module. For this module, we transitioned the conceptual framework we used from the CRAAP (Currency, Relevance, Authority, Accuracy, and Purpose) test [19] to the lateral reading approach [20,21]. Lateral reading follows the mnemonic SIFT: Stop, Investigate the source, Find trusted coverage, and Trace claims to the original context. We switched to lateral reading/SIFT because it is more evidence-based and reflects the methods used by professional fact-checkers [22]. We added a new module on day three to provide more information about careers in librarianship, which in year one had been minimal. This module was prompted by student interest in various job types and the salaries of librarians. After the didactic portion of each module, we increased the amount of time allocated for students to work on the corresponding section of their final projects, in part by removing the viewing of Advisory Board videos from classroom time.

**Table 2:** Changes to the Curriculum Between Years One and Two

Module Number	Year One	Year Two
0	Introduction & Pretest	Introduction & Pretest
1	Find and Identify Credible Health Information	Credible Information and Disease Research
2	Health Literacy	Career Introduction
3	Misinformation & Disinformation	Find and Identify Credible Health Information*
4	How To Read News Articles	Health Literacy and Misinformation
5	Citing Credible Information Resources	Citing Credible Information Resources

* In Year Two, Year One modules 2 and 3 were merged into the new module 4.

Another key change was to the program sites. Due to the retirement of the teacher who hosted us at TMA in year one, for year two, another teacher on our team (JB) offered to host the course in her anatomy class at Knowledge and Power Preparatory Academy International High School (KAPPA), a New York City public school in the Bronx, with MSMA continuing as the second site. Like TMA and MSMA, KAPPA’s student body is majority non-white (69% Hispanic) [23].

We revised the pre-posttest and the final project assessment rubric to reflect these changes and the revised learning outcomes. We quizzed students on the pre/posttest questions throughout the curriculum to help them prepare. We emphasized to students before they took the posttest that their answers were important to demonstrate what they had learned. As in the previous year, the posttest did not impact their grade.

### Year Two Assessment Methods

The year two assessment methods were the same as those used in year one. The revised pre-posttest consisted of ten multiple-choice questions (two per module) assessing course and session-level learning outcomes, administered via a Qualtrics form on the first (pretest) and last (posttest) days of class. We conducted an unpaired t-test to assess statistical significance in the score change between the pre- and posttests. Two MSMA students completed the pretest but did not attend class the day the posttest was administered. Since the MSMA pretest had been anonymous, it was impossible to remove these two students from the evaluation and it was not possible to conduct a paired t-test. See Table Three for the questions and the Supplementary File on the Open Science Framework for the answer choices [24].

**Table 3:** Year Two Pre-Posttest Questions

Question Number	Question Text
Q1	Juan struggles with depression. His doctor has prescribed him a drug to help him manage it, but Juan is unhappy with its side effects. While researching other antidepressants, Juan reads an article about a newly approved drug on a website he’s unfamiliar with. Which of the following will best help him evaluate the credibility of the article’s claims about the drug?
Q2	Librarians work in many fields. The role of a health sciences librarian is to:
Q3	Which of the following is most accurate? *(Answer choices assessed the educational requirements for health sciences librarians)*
Q4	Which of the following best defines health literacy?
Q5	An account you follow on TikTok shares a video where they interview a famous doctor about a special diet they are selling to help people shed fat quickly while adding muscle. Which of the following is most true?
Q6	Which statement best defines misinformation?
Q7	Which of the following is most true of MedlinePlus?
Q8	Popular literature typically has which of the following characteristics?
Q9	In addition to the URL/Web site address, what would a citation need to include to make an online news article findable?

The focus groups took place after the last day of class and were administered by MIM volunteers using a standard script (see Supplementary File). Moderators recorded the audio of posttest focus groups. Note-takers summarized responses. A member of the study team (AH) subsequently transcribed recordings by hand. We conducted thematic analysis of focus group transcripts following the methods developed by qualitative psychologists Virginia Braun and Victoria Clarke [25].

Data from the Year Two pre-posttest and focus groups are available in the Supplementary File [24].

### Year Two Assessment Results

44 students completed the curriculum across the two schools. The mean student score improved from 5.77 on the pretest to 7.60 posttest; this improvement was statistically significant (p < 0.0001). Table Four shows performance by question for both schools.

**Table Four:** Year Two Pre-Posttest Scores by Question

Question	MSM pretest # correct responses (N=21)	MSM pretest percentage correct (N=21)	MSM posttest # correct responses (N=19)	MSM posttest percentage correct (N=19)	KAPPA pretest # correct responses (N=23)	KAPPA pretest percentage correct (N=23)	KAPPA posttest # correct responses (N=23)	KAPPA posttest percentage correct (N=23)
Juan struggles with depression. His doctor has prescribed him a drug to help him manage it, but Juan is unhappy with its side effects. While researching other antidepressants, Juan reads an article about a newly approved drug on a website he’s unfamiliar with. Which of the following will best help him evaluate the credibility of the article’s claims about the drug?	9	43%	18	95%	12	52%	20	87%
Librarians work in many fields. The role of a health sciences librarian is to?	14	67%	11	58%	16	70%	23	100%
Which of the following is most accurate?	4	19%	5	26%	4	17%	14	61%
Which of the following best defines health literacy?	19	90%	18	95%	18	78%	22	96%
An account you follow on TikTok shares a video where they interview a famous doctor about a special diet they are selling to help people shed fat quickly while adding muscle. Which of the following is most true?	14	67%	17	89%	21	91%	22	96%
Which statement best defines misinformation?	14	67%	13	68%	19	83%	20	87%
Which of the following is most true of MedlinePlus?	13	62%	18	95%	23	100%	23	100%
Popular literature typically has which of the following characteristics?	7	33%	13	68%	14	61%	23	100%
In addition to the URL, what would a citation need to include to make an online news article findable?	15	71%	19	100%	18	78%	20	87%

We identified two themes and three sub-themes from our thematic analysis of the focus group data. The first theme, “Assessing outcomes,” extended and helped provide context for the assessment of learning outcomes we conducted through the pre/posttest and the final project, and was further divided into three sub-themes. In sub-theme 1.1, “Understanding the profession of librarianship,” students demonstrated knowledge recall about the different types of librarianship and about health sciences librarianship in particular. In sub-theme 1.2, “Career interests,” students told us about themselves, their preferences, and their career goals. In general, while students saw librarians’ work as valuable, most students were not interested in pursuing a career as a librarian. Sub-theme 1.3, “Information skills,” provided evidence of student learning in terms of their information skills, consistent with our findings from the pre- and posttest and final project grading. They demonstrated knowledge recall particularly in the areas of evaluating sources, defining and using MedlinePlus, and distinguishing between scholarly and popular literature.

In theme two, “Student feedback about the course,” students generally stated that they enjoyed the course and the activities, in particular the performance task and researching celebrity health topics. Some students enjoyed designing their slide presentations, presenting, and watching their classmates’ presentations. Students least enjoyed creating and formatting references for their project. An aspect of the final project they reported as particularly challenging was finding claims for their topic and evaluating them, which instructors had also observed during class. Future instructors may want to allocate more time for this or consider pre-selecting examples and providing them to students to simplify and make things clearer. Students identified public speaking and group work as challenging aspects of the final project. Most high school students have limited experience with the former; for the latter, they described navigating group decision-making as one of the challenges. While these were not core learning outcomes for the curriculum, they helped the students build transferable skills, preparing them for future coursework and eventually the workplace.

In addition to helping us assess the curriculum, the focus groups served as an informal learning reinforcement activity by asking students to recall what they learned, describe it in their own words, engage in peer discussion and learning, and reflect on their understanding and integrate it into their individual knowledge and experiences.

While not formally part of our assessment approach, the students’ final group project presentations provide an informal third data point. We scored their final project presentations using an updated scoring rubric, available in our online toolkit [26]. The rubric contained five domains: misinformation in popular media; disease content knowledge; evaluation of a source using SIFT; citations and references in APA format; and presentation skills and quality. Teams could receive up to four points per domain, with four points representing “Above standard,” three “meets standard,” two “approaches standard,” and one “below standard,” for a maximum total of twenty points. The scores from the librarian instructors and teacher were averaged to produce a composite score for each group. For the final project presentations, average scores by school were 16.7 at MSMA and 15.1 at KAPPA, both translating to “Meets standard” on the scoring rubric.

### Dissemination

We have published the curriculum online under the Creative Commons Attribution - NonCommercial-NoDerivatives 4.0 International License (CC BY-NC-ND 4.0) [18], to allow for its reuse and customization by other organizations. On June 18, 2025, we presented a virtual Train the Trainer webinar on how to implement the curriculum, sponsored by Region 7 of the United States Network of the National Library of Medicine [27].

## DISCUSSION

Our project provides lessons for other librarians seeking to implement our curriculum or to develop their own in high school classrooms. We found the involvement of the experienced high school teacher (JB) on our curriculum development team invaluable, both in navigating the logistics of the school settings and in designing an effective and engaging curriculum. Classroom technology also proved to be an important variable, as we found it varied significantly across our sites and sometimes posed a barrier. Organizations planning to implement the curriculum in other settings must familiarize themselves with the classroom technology in the schools they partner with, as well as any constraints, during the planning phase.

Our ability to draw conclusions about student learning is limited by the fact that we did not use a validated health information literacy tool. Future research could evaluate the curriculum’s effectiveness using one or more of the many validated assessment tools. As previously noted, we did not collect the student’s names in the year two pre-test at one school, meaning that we could not conduct a paired t-test nor could we remove students who failed to take either the pre- or posttest from the sample at that school. We also encountered some limitations with our focus group format. Response “herding” was common. After the first student answered a question, students often tended to paraphrase their response or focus on whatever themes the first student had mentioned. Another limitation of the focus group format was that we relied on MIM volunteers who were not trained in administering focus groups to lead these discussions. It is possible that some of the response herding could have been mitigated by more experienced facilitators.

Finally, we designed and implemented our curriculum in 2023-2024 at the end of the Biden administration. The second Trump administration, which began in January 2025, has subsequently made significant changes to health information resources such as MedlinePlus. For example, as of October 2025, the “Health Disparities” article on MedlinePlus included a message at the top of the page stating that “information on this page promoting gender ideology is extremely inaccurate and disconnected from truth… This page does not reflect reality and therefore the Administration and this Department reject it” [28]. While our curriculum presents government information as neutral and objective, it has since become apparent the way such information is selected and presented can represent political agendas. We encourage instructors to consider this when choosing which resources to recommend to students.

This case study demonstrates that students introduced to health sciences librarianship in high school are able to understand the work of the profession, discuss questions they have about the profession, and engage in basic health literacy activities. In our focus groups, some of the students reported little or no experience with libraries, thus not giving them as much exposure to build information literacy skills or consider health sciences librarianship as a career. Reuse of the curriculum in other communities will provide students from diverse and underrepresented communities the chance to develop these skills. While students were generally still not interested in a career as a librarian immediately after completing the course, it could be valuable to track students’ interest over time (e.g. surveying them after several years as to their eventual career choice).

Future research may focus on implementing the curriculum in other settings and with other populations. We encourage any group who would like to use this curriculum to tailor it to their audience for optimal engagement, and to consider revising it to incorporate one or more of the validated health information literacy assessment tools that are currently available.

## Data Availability

Data associated with this article are available on the Open Science Framework [24].
